# Effects of a Single Proprioceptive Neuromuscular Facilitation Stretching Exercise With and Without Post-stretching Activation on the Muscle Function and Mechanical Properties of the Plantar Flexor Muscles

**DOI:** 10.3389/fphys.2021.732654

**Published:** 2021-09-14

**Authors:** Marina Reiner, Markus Tilp, Gaël Guilhem, Antonio Morales-Artacho, Masatoshi Nakamura, Andreas Konrad

**Affiliations:** ^1^Institute of Human Movement Science, Sport and Health, University of Graz, Graz, Austria; ^2^Laboratory Sport, Expertise and Performance, French Institute of Sport (INSEP), Paris, France; ^3^Department of Physical Therapy, Niigata University of Health and Welfare, Niigata, Japan

**Keywords:** proprioceptive neuromuscular facilitation stretching, post-stretching activation, specific warm-up, muscle function, muscle shear modulus, muscle stiffness, mechanical properties, plantar flexor muscles

## Abstract

A single proprioceptive neuromuscular facilitation (PNF) stretching exercise can increase the range of motion (ROM) of a joint but can lead to a decrease in performance immediately after the stretching exercise. Post-stretching activation (PSA) exercises are known as a possible way to counteract such a drop in performance following a single stretching exercise. However, to date, no study has investigated the combination of PNF stretching with PSA. Thus, the aim of this study was to compare the effects of a PNF stretching exercise with and without PSA on the muscle function (e.g., ROM) and mechanical properties of the plantar flexor muscles. Eighteen physically active males volunteered in the study, which had a crossover design and a random order. The passive shear modulus of the gastrocnemius medialis (GM) and gastrocnemius lateralis (GL) was measured in a neutral position with shear wave elastography, both pre- and post-intervention. Maximum voluntary isometric contraction (MVIC) peak torque, maximum voluntary dynamic contraction peak torque, dorsiflexion ROM, and passive resistive torque (PRT) were also measured with a dynamometer. The interventions were 4×30s of PNF stretching (5s of contraction) and two sets of three exercises with 20 or 40 fast ground contacts (PNF stretching+PSA) and PNF stretching only. ROM was found to have increased in both groups (+4%). In addition, the PNF stretching+PSA group showed a decrease in PRT at a given angle (−7%) and a decrease in GM and mean shear modulus (GM+GL; −6%). Moreover, the MVIC peak torque decreased (−4%) only in the PNF stretching group (without PSA). Therefore, we conclude that, if PNF stretching is used as a warm-up exercise, target-muscle-specific PSA should follow to keep the performance output at the same level while maintaining the benefit of a greater ROM.

## Introduction

Stretching is a commonly used technique during a warm-up protocol. Several studies have compared different stretching techniques and suggested that proprioceptive neuromuscular facilitation (PNF) and static stretching (SS) can be equally effective for increasing the range of motion (ROM; Konrad et al., 2017; Lempke et al., 2018). However, others (Miyahara et al., 2013) have reported a favorable effect for PNF stretching compared to SS. During SS, the target muscle is passively stretched at the end ROM, while PNF stretching includes a contraction phase of the target muscle in the stretched position. The most common PNF methods comprise “contract and relax” or “contract relax and antagonistic contract” procedures (Sharman et al., 2006). The changes in ROM following PNF stretching might be associated with the observed decreased passive resistive torque (PRT) at the same joint angle as pre-intervention (Kay et al., 2015; Konrad et al., 2017). Differences in the torque-angle curve of the PRT indicate a decrease in the soft tissue component of the muscle (e.g., muscle stiffness: Konrad et al., 2017; tendon stiffness: Kay et al., 2015). This mechanism is one possible explanation for the changes in ROM. Moreover, Nakamura et al. (2015) found a higher end ROM after PNF stretching and concomitant higher passive peak torque values at the maximal joint position. This finding indicates an increased tolerance to stretch (e.g., less pain sensitivity, Magnusson et al., 1996).

Functional performance parameters have often been measured after a single bout of PNF stretching. However, there are differences between strength parameters, such as maximum voluntary isometric contraction (MVIC) peak torque, and muscle power parameters, such as jump height or sprint time. Several studies have reported a decrease of MVIC peak torque (Marek et al., 2005; Bradley et al., 2007; Konrad et al., 2017) or muscle power parameters (Bradley et al., 2007) after a single PNF stretching exercise, while other studies have found no such acute changes in functional performance (Christensen and Nordstrom, 2008; Manoel et al., 2008; Molacek et al., 2010). Pacheco et al. (2011) even observed an increase in muscle power parameters (counter movement jump, squat jump, and drop jump) after a single bout of PNF stretching. However, such an increase in performance is rare, and a review of 19 studies of PNF stretching reported a mean performance impairment of 5.5% following a single PNF stretching exercise (Behm et al., 2016).

A possible way to preserve the gain in ROM due to stretching while counteracting possible performance impairment following a single stretching exercise might be target-muscle-specific post-stretching activation (PSA). Samson et al. (2012) found an increase in sprint speed after the combination of stretching (static or dynamic) and PSA (high knee skipping, high knee running, and butt kick running; done twice for 20m), but no change if the stretching was combined with a general warm-up routine (5min of running on a 200m track at 70% of the individual’s age-predicted heart rate). Moreover, in combination with PSA, static stretching was found to be more efficient for the sit-and-reach ROM test than dynamic stretching. Another study by Reid et al. (2018) reported a favorable effect in functional performance parameters (e.g., strength or jumps) following several stretching conditions (30s, 60s, 120s, and no stretching) with PSA (30s each of gluteal kicks and high knees, and 60s each of walking hip openers, dynamic leg kicks to opposing hand, walking lunges with rotation, and the inchworm), compared to the same stretching conditions without PSA. In contrast, Blazevich et al. (2018) found no changes in ROM and functional performance parameters (sprint running, vertical jumping, and change in direction) after a short duration static (5s and 30s) or dynamic (5rep) stretching intervention within a warm-up, even though the static stretching was performed at the point of discomfort and the maximum ROM was reached during the dynamic stretching.

In their recent review, Behm et al. (2021) determined that changes in normalized muscular activity [electromyography (EMG)/ M wave] are a likely explanation for performance impairments after prolonged static stretching. Further possible mechanisms mentioned are a reduction in persistent inward current flow in the dendritic regions of motoneurons and changes in exteroceptive reflex (Behm et al., 2021). However, more research is needed to clarify the mechanism behind the force loss following stretching (Behm et al., 2021). There is a consensus in the literature (Behm et al., 2016) that the stretching-induced impairments in functional performance parameters could be reversed to baseline with PSA, or it may even be possible to improve functional performance with PSA. Although this has been shown in static and dynamic stretching, to date, no study has investigated the acute effects in functional performance parameters, such as strength of PNF stretching combined with PSA.

The mechanistic basis of PNF effects on ROM and performance parameters may be partially explained by changes in muscle-tendon unit mechanical properties (e.g., muscle stiffness). By combining torque values and the displacement of the muscle-tendon junctions in the plantar flexors [i.e., gastrocnemius medialis (GM)] during isometric contractions, Konrad et al. (2017) and Kay et al. (2015) observed a decrease in active muscle and muscle-tendon (joint) stiffness after a single PNF stretching intervention. In addition, Kay et al. (2015) observed decreased passive tendon stiffness after a single bout of PNF stretching. Although the applied technique is aimed at assessing muscle and tendon stiffness (e.g., Konrad and Tilp, 2020), many more structures, such as fascia, nerve, and skin, affect the measurements (Weppler and Magnusson, 2010). In contrast to the method of calculating the stiffness in a muscle-tendon unit, shear wave elastography (SWE) can assess the local stiffness of individual muscles (Reiner et al., 2021), which might be one way to explain the direct effects of PNF stretching with or without PSA on muscle mechanical properties. To the best of our knowledge, to date, no studies have analyzed local muscle stiffness with SWE after a PNF stretching intervention in young adults.

The aim of this study was to compare the effects on the plantar flexor muscles of a single PNF “contract and relax” stretching intervention for 2min (PNF stretching) with the effects of the same intervention combined with PSA (PNF stretching+PSA). For both groups, we hypothesized an increase in ROM and a decrease in PRT and muscle shear modulus. Moreover, we further assumed a decrease in MVIC peak torque for the PNF stretching group, but no change for the PNF stretching+PSA group.

## Materials and Methods

### Experimental Design

**Figure 1:**
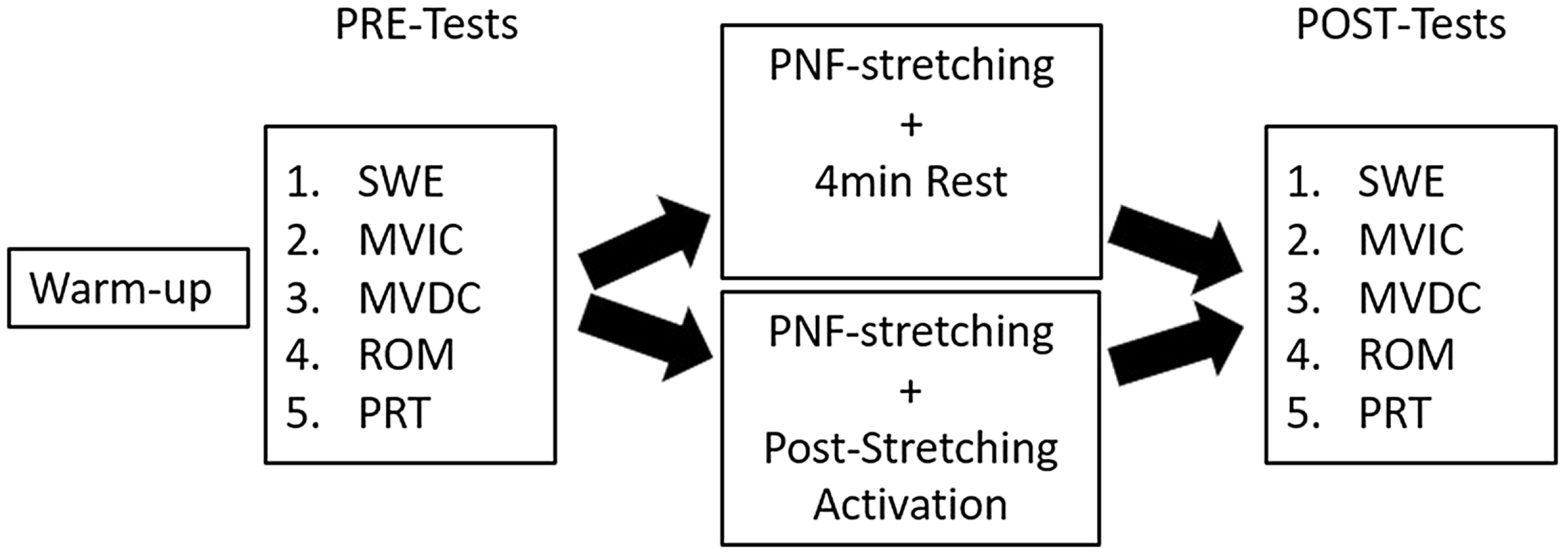
Overview of the test procedure. SWE, shear wave elastography; MVIC, maximum voluntary isometric contraction; MVDC, maximum voluntary dynamic contraction; ROM, range of motion; PRT, passive resistive torque; control condition=4min rest; and post-stretching activation (PSA)=2×(skipping with 40 ground contacts, 20 jumps with straight legs, and high knee running with 40 ground contacts).

All participants were asked to visit the laboratory on two separate days to complete both interventions (PNF stretching+PSA vs. PNF stretching alone). The appointments were separated by 48h, and the participants visited the laboratory at the same time of the day±1h. Each participant chose a hidden card to randomize the interventions. Both test days started with a 10min warm-up routine on a stationary bike (Monark, Ergomedic 874 E, Sweden) at 60rev.min^−1^ (Kay et al., 2015) and 60W, prior to the test procedure. ROM, MVIC peak torque, maximum voluntary dynamic contraction (MVDC) peak torque, PRT, and passive muscle shear modulus (i.e., as an index of passive stiffness) of the right plantar flexor muscles [GM and gastrocnemius lateralis (GL)] were examined both pre- and 2min post-intervention. The tested muscle function parameters were plantar flexor MVIC peak torque, MVDC peak torque, PRT of the triceps surae muscles, and ankle dorsiflexion ROM. Tests were performed in the order listed in Figure 1, while the order for the SWE assessment was GM followed by GL.

### Participants

An *a priori* sample size calculation (primary outcome variable: ROM) for a repeated-measures ANOVA based on the literature (Phillips et al., 2018) suggested a necessary group size of 15 subjects (*α*=0.05 and *β*=0.8, *f*=0.4). Therefore, we selected 18 healthy, physically active male participants (age: 26.5±4.2years; body mass: 81.6±8.4kg; and height: 184.8±8.3cm) as volunteers in this study, who visited the laboratory on two separate days. All study volunteers were free of any injuries of the lower extremities. Participants were asked not to perform any exhausting exercise in the 72h prior to the appointments. The participants were also informed about the test procedures and provided written consent. The study was approved by the ethical commission of the University of Graz (approval code GZ. 39/68/63 ex 2020/21), and the study design conformed to the standards of the Declaration of Helsinki.

### Procedures

#### Muscle Shear Modulus

Muscle shear modulus was measured on the GM and GL by SWE with an ultrasound scanner (Aixplorer V6, Supersonic Imaging, Aix-en-Provence, France). A linear transducer array (4–15MHz, SuperLinear 10-2; Vermon, Tours, France) was coupled to the machine and used in SWE mode (musculoskeletal preset, penetration mode, smoothing level 5, persistence off, and scale 0–300kPa). A handheld technique was used, allowing a reliable measurement of the muscle shear modulus (Bercoff et al., 2004; Lacourpaille et al., 2012; Hug et al., 2015). The participant was positioned prone on the dynamometer, with hip and knees fully extended (180°: full knee and hip extension). The ankle angle was set at anatomical zero (=90°). The participant wore no shoes, and a custom-made laser device was used to align the center of rotation of the dynamometer with the ankle joint axis. To ensure the same assessment position on the dynamometer in both appointments, the exact positions of the adapter and motor of the dynamometer were recorded after the first positioning. To ensure an identical probe placement in all measurements, the same procedure (reusable foil and B-mode picture) was used as described in Reiner et al. (2021). The reusable foil was placed on the participant’s calf and birthmarks and scars were marked with a permanent marker for a proper repositioning of the foil. Moreover, the measuring position of the probe was marked as well to find the same spot throughout the SWE measurements. A B-Mode picture from the baseline assessment on a second screen was supportive to place the probe on the same spot during all trials. SWE was performed in the same order in all measurements: GM followed by GL. Shear modulus of the GM and GL was measured around the proximal third between the calcaneus and the popliteal fossa medial and lateral, respectively. Tissue and structure deformation was avoided by applying as little pressure as possible to the skin (Kot et al., 2012). The transducer was held in place and aligned in plane with the muscle fascicles during the whole measurement (Le Sant et al., 2017). The range of interest (ROI) was positioned centrally and maximized as much as possible, but excluding any aponeurosis. Prior to the SWE, pre-measurement conditioning was performed to ensure the same muscle conditions during the shear modulus testing. Therefore, the ankle was moved passively for 5cycles from 20° plantar flexion to 20° dorsiflexion with an angular velocity of 5°/s. Participants were asked to relax completely during the whole process. For each muscle, three videos of 15s each were recorded. The mean of the five consecutive frames with the lowest standard deviation of the shear modulus averaged over the ROI within a video was considered for further analysis. The two closest mean values per muscle from the three videos taken for each muscle were used to calculate the mean passive stiffness per muscle (Morales-Artacho et al., 2017). Furthermore, the mean SWE values of the GM and GL were also calculated.

#### Maximum Voluntary Isometric Contraction Peak Torque

The MVICs were performed on an isokinetic dynamometer (Con Trex MJ, CMV AG, Dübendorf, Switzerland). The participant was positioned prone on the dynamometer, with knee and hip angle fully extended (as explained before). The participant’s trunk, hip, and foot were fixed with straps to minimize position changes due to evasive movements. Two MVICs of 5s each were performed, separated by 1min. Participants were asked to relax their arms during the measurement and received strong verbal encouragement while pushing as hard as possible. The attempt with the highest peak torque value was considered for further analysis.

#### Maximum Voluntary Dynamic Contraction Peak Torque

The MVDCs were performed in the same position as the MVIC trials. The foot adapter moved in a range of 20° plantar flexion to 20° dorsiflexion for 3cycles with a velocity of 60°/s. The participant was asked to stay relaxed when the foot was moved into dorsiflexion but to push as hard as possible against the plate while moving into plantar flexion. The participant’s arms should stay relaxed during the whole measurement. Two trials were performed, and the one with the higher average of the two highest peak values within the 3cycles was considered for further analysis.

#### Range of Motion

**Figure 2:**
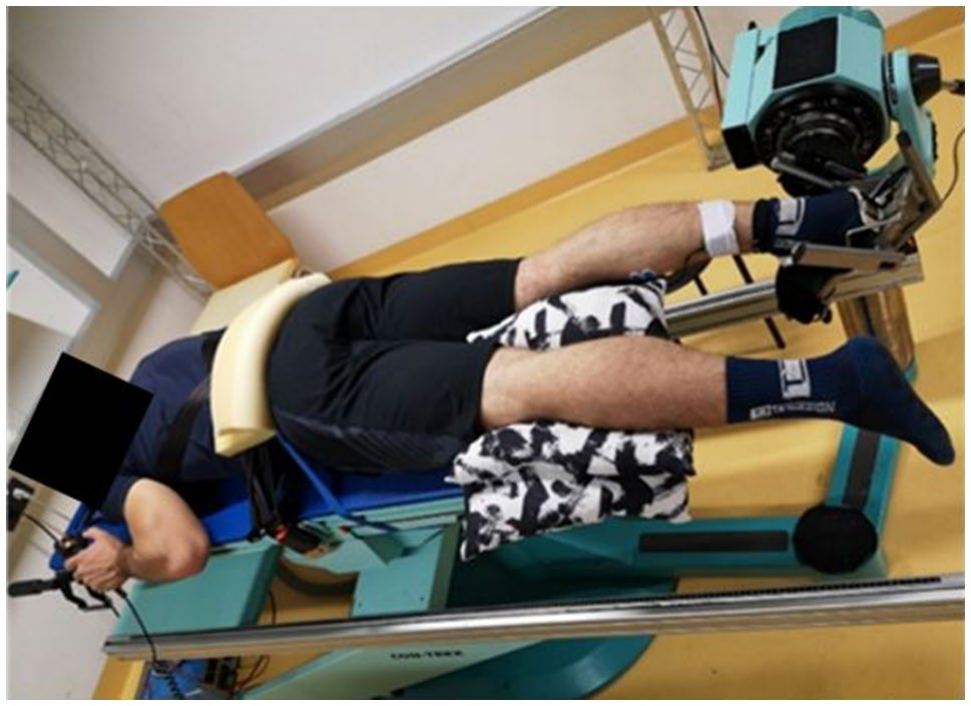
Measurement setup in the laboratory. The participant is positioned prone and fixed on the dynamometer to prevent evasive movements. The pillow underneath the non-testing leg was used to increase subjects’ comfort during the measurements.

The positioning for the ROM measurement was the same as that for the MVIC trial. Participants were asked to completely relax. The starting position was 90° in the ankle joint (neutral position), and participants were asked to operate the dynamometer by remote control on their own to get into the maximum possible dorsiflexion position, until a maximum tolerable stretch was reached (Figure 2). The dynamometer’s angular velocity was set to 5°/s. The difference between neutral position and the maximum dorsiflexion angle was recorded and defined as the dorsiflexion ROM (Konrad et al., 2017).

#### Passive Resistive Torque

The PRT measurement was made immediately after the ROM measurement. The foot plate was moved for 5cycles from 20° plantar flexion to maximum dorsiflexion (as assessed in the previous ROM assessment), with an angular velocity of 5°/s. The velocity was set to 5°/s to exclude any reflective muscle activity (Kubo et al., 2002; Mahieu et al., 2009). The participant was asked to relax completely. For further analysis, the cycle with the lowest torque value of the last 3cycles in the dorsiflexion phase was taken for further analysis (PRT_max_). Furthermore, the values of the cycle with the lowest torque value of the last 3cycles in the dorsiflexion phase were compared at the same angle reached in both measurements, i.e., pre- and post-intervention (PRT at a given angle).

#### Surface Electromyography

Muscle activity was monitored using EMG (myon 320, myon AG, Zurich, Switzerland) during the SWE, MVIC, and PRT measurements. Skin preparation and placement of the surface electrodes (Blue Sensor N, Ambu A/S, Ballerup, Denmark) on the medial part of the GM were performed according to SENIAM recommendations (Hermens et al., 1999). The signal was monitored during the measurement, and if signal changes were detected visually during the passive trial, the measurement was repeated. For the PRT and SWE, if minimal changes in the raw EMG signal could be detected during the analysis process, a *post-hoc* analysis was performed to ensure that the subject was relaxed enough, i.e., did not show EMG activity exceeding 5% of MVIC (Gajdosik et al., 2005; Kato et al., 2010). For the *post-hoc* analysis, the EMG signal was high-pass filtered (10Hz, Butterworth). The root-mean square (RMS, 50ms moving window) values were also calculated and were compared to the peak EMG amplitude during the MVIC.

#### Interventions

**Figure 3:**
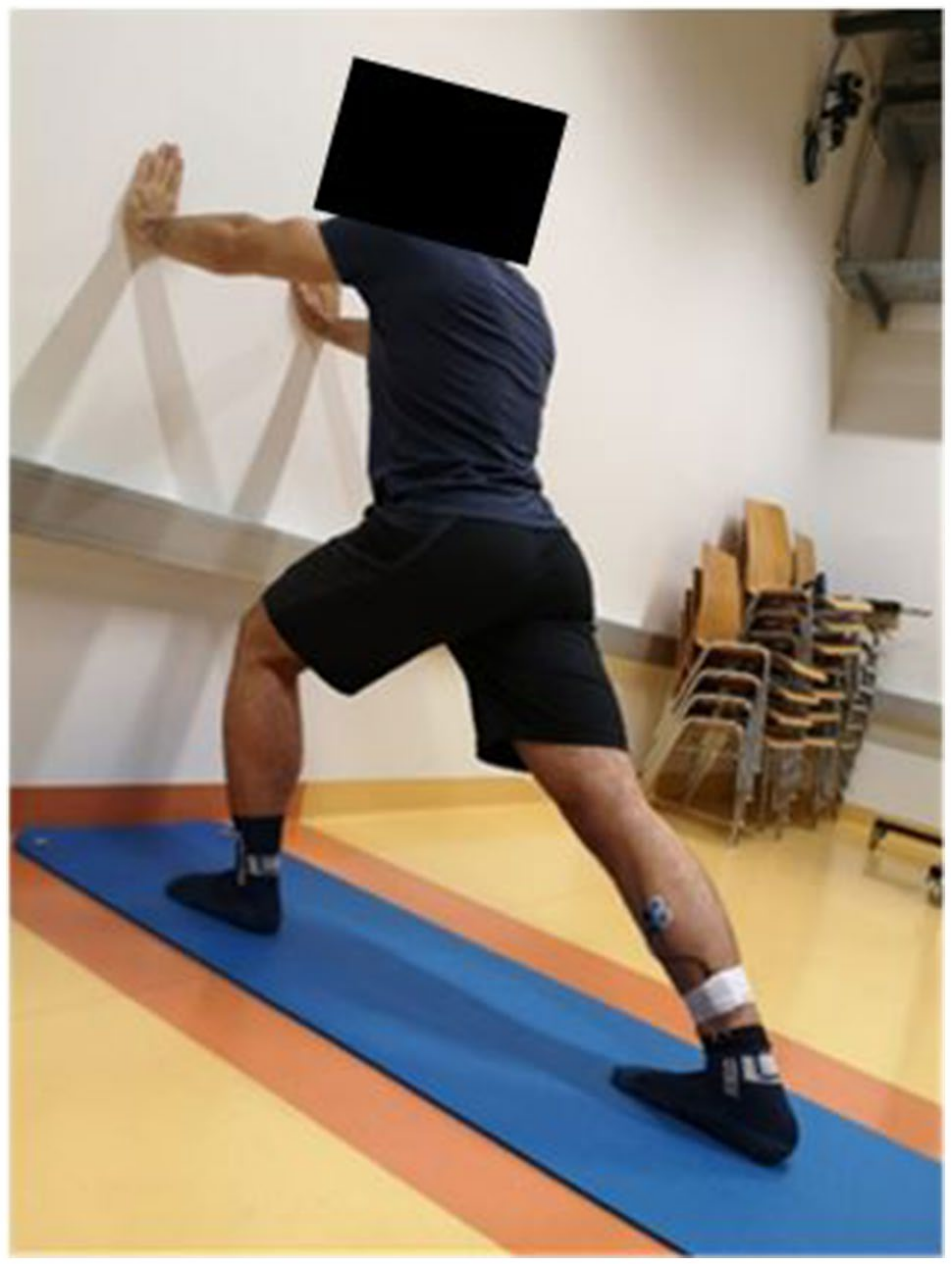
The standing wall push exercise used for the proprioceptive neuromuscular facilitation (PNF) stretching intervention.

For the PNF stretching of the plantar flexors, the “standing wall push” exercise was chosen. A slip-free mat was placed on the ground to ensure proper foot placement in the stretching position. The participant was asked to stand upright in front of the wall, with both hands on the wall at the height of the chest. After the start command, the test leg was moved with an extended knee behind the body. The toes of both feet were front facing, and the leg was placed with the heel touching the ground to receive a stretch until the point of discomfort (Figure 3). This stretching intensity was maintained for 25s, followed by maximal contraction of the plantar flexors in the stretching position for 5s. This procedure was repeated four times, separated by 10s breaks. Overall, the PNF stretching duration was 2min. This duration was chosen to likely induce changes in performance at least when applied without PSA (Behm and Chaouachi, 2011; Behm et al., 2016, 2021).

After the PNF stretching, the participant either had a break of 4min walking around the laboratory (PNF stretching) or performed the PSA (PNF stretching+PSA) next to dynamometer. For the PSA, the participant was asked to perform three different exercises as fast as possible: skipping with 40 ground contacts, 20 jumps with straight legs, and high knee running with 40 ground contacts. The PSA exercises were performed twice in the order described, with a 30s break between each exercise. A stopwatch was used for the break management, and the ground contacts were counted by the investigator who also supervised the stretching session.

### Statistical Analysis

SPSS (version 26.0, SPSS Inc., Chicago, Illinois) was used for all the statistical analyses. To determine the intra-rater and inter-day reliability of the SWE measurements, intraclass correlation coefficients (ICC, 2-way mixed-effect model, and absolute agreement definition) were used. Furthermore, the standard error of the measurement of the shear modulus values was also calculated.

The variables tested were ankle dorsiflexion ROM, PRT, MVIC peak toque, MVDC peak torque, shear modulus of the GM and GL, and mean shear modulus of the GM+GL. Shapiro-Wilk test confirmed the normal distribution of all the variables. A two-way repeated-measures ANOVA [factors: time (pre vs. post)] and intervention (PNF stretching vs. PNF stretching+PSA) were performed. If ANOVA with repeated measures was significant, a *t*-test was performed between the pre- and post-values of the parameters of each group. To test if the baseline values in both conditions were similar, paired *t*-tests were performed between the pre-values of a parameter. To test for possible differences between the two conditions (PNF stretching vs. PNF stretching+PSA), paired *t*-tests of the delta values (post–pre) of each parameter in both groups were performed. Correlation analyses were performed between the delta (post–pre) values of PRT_max_ and ROM, and between the delta (post–pre) values of ROM and the shear modulus of the GM and GL and were assessed with Pearson correlation coefficients. Cohen’s *d* was calculated following the suggestions of Cohen (2013). Thus, the effect size *d* was defined as 0.2, 0.5, and 0.8 for a small, medium, and large effect size, respectively. The *α* level was set to 0.05.

## Results

### SWE Reliability and Baseline Measurement Quality

The SWE ICC values between the pre-measurements of both test days (PNF stretching vs. PNF stretching+PSA) for the GM and GL were 0.77 and 0.89, respectively. Moreover, the standard errors of the measurement for the GM and GL were 0.71kPa and 0.48kPa with a 95% confidence interval of 0.45–0.91 and 0.73–0.96, respectively. Baseline characteristics for pre-measurements on both test days showed no significant difference in GM shear modulus (*p*=0.28), GL shear modulus (*p*=0.32), mean shear modulus of the GM+GL (*p*=0.842), MVIC peak torque (*p*=0.37), MVDC peak torque (*p*=0.79), ROM (*p*=0.87), PRT (*p*=0.56), and PRT at a given angle (*p*=0.951).

### Shear Modulus Values

**Figure 4:**
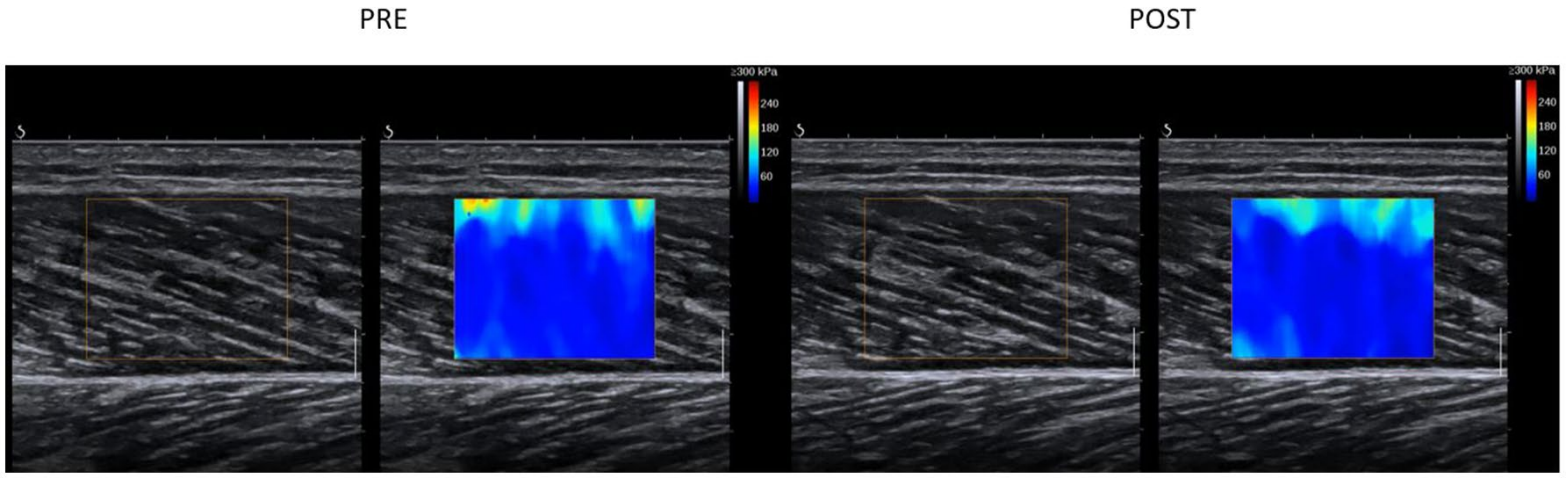
An example for signal changes in shear wave elastography in the GM muscle in the PNF+PSA group. For the analysis, the area within the colored box was analyzed. Each colored pixel has a value according to the scale on the right upper corner (max 300kPa) and the average of the chosen area was taken for further analysis.

The ANOVA test revealed a significant time effect for the shear modulus of the GM (*p*=0.03; *F*=5.65; *r*=0.5; *df*=17), but no group effect (*p*=0.84; *F*=0.04; *r*=0.05; *df*=17) or group×time interaction effect (*p*=0.06; *F*=4.16; *r*=0.44; *df*=17). A pairwise comparison of the shear modulus values of the GM showed a significant decrease in the PNF stretching+PSA group (*p*=0.008; *d*=−0.71, medium effect), but not in the PNF stretching group (*p*=0.71; *d*=−0.09; Figure 4; Table 1).

**Table 1:** Results for the maximum voluntary isometric contraction (MVIC), maximum voluntary dynamic contraction (MVDC), dorsiflexion range of motion (ROM) of the ankle joint, passive resistive torque (PRT), PRT at a given angle, shear modulus values for the GM and GL, and the mean shear modulus of the GM+GL of both groups, i.e., PNF stretching+PSA (left) and PNF stretching alone (right).

	PNF+PSA						PNF only						% change	
	PRE			POST			PRE			Post			PNF+PSA	PNF only
	mean	±	SD	mean	±	SD	mean	±	SD	mean	±	SD		
MVIC (Nm)	158.7	±	28.9	154.3	±	25.7	156.3	±	26.8	151.6^*^	±	28.0	−3%	−4%^*^
MVDC (Nm)	157.8	±	25.2	165.6	±	29.6	159.2	±	30.4	159.4	±	29.0	3%	0%
ROM (°)	40.1	±	6.8	42.1^*^	±	8.0	40.2	±	7.1	42.3^*^	±	8.1	4%^*^	4%^*^
PRT_max_ (Nm)	53.2	±	14.1	56.2	±	17.2	54.1	±	17.9	57.1	±	17.5	1%	4%
PRT° (Nm)	49.9	±	13.9	47.2^*^	±	13.6	50.1	±	18.2	49.8	±	15.8	−7%^*^	0%
Shear modulus GM (kPa)	9.8	±	1.7	9.2^*^	±	1.5	9.5	±	1.3	9.4	±	1.4	−7%^*^	−2%
Shear modulus GL (kPa)	7.4	±	1.6	7.1	±	1.6	7.7	±	1.4	7.7	±	1.5	−5%	0%
Mean Shear modulus GM+GL (kPa)	8.6	±	1.4	8.2^*^	±	1.3	8.6	±	1.2	8.6	±	1.2	−6%^*^	−1%

* =significant difference between pre- and post-measurement data, mean±*SD*.

MVIC, maximum voluntary isometric contraction peak torque; MVDC, maximum voluntary dynamic contraction peak torque; ROM, dorsiflexion range of motion; PRT_max_, passive resistive torque at maximum dorsiflexion; and PRT, passive resistive torque at a given angle.

For the shear modulus of the GL, the ANOVA test revealed a significant group effect (*p*=0.04; *F*=5.06; *r*=0.48; *df*=17), but no time effect (*p*=0.33; *F*=1.02; *r*=0.24; *df*=17) or group×time interaction effect (*p*=0.09; *F*=3.18; *r*=0.4; *df*=17). A pairwise comparison of the shear modulus of the GL showed no significant changes in any group, i.e., PNF stretching (*p*=0.81; *d*=0.58) and PNF stretching+PSA (*p*=0.07; *d*=−0.46; Table 1).

**Figure 5:**
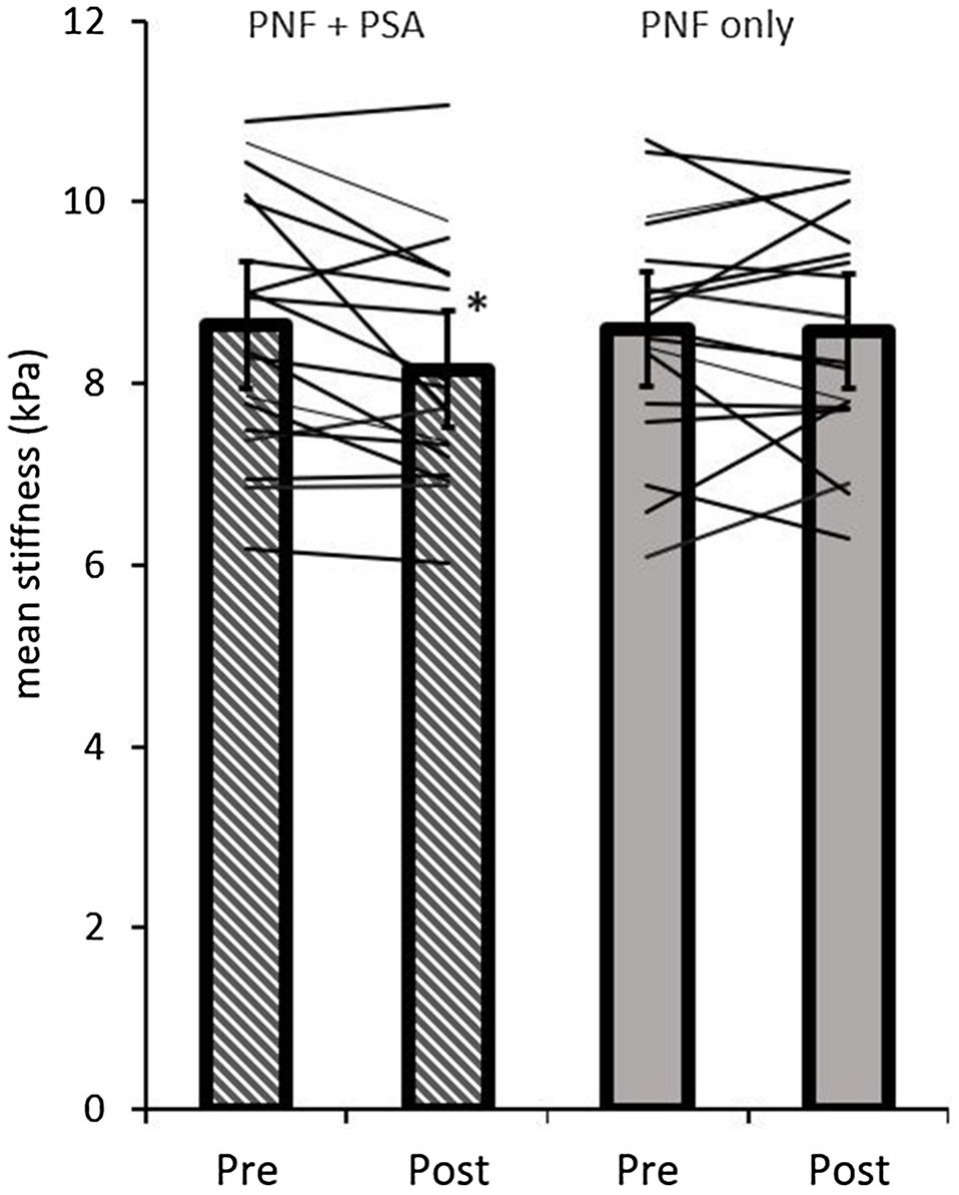
Pre- and post-mean shear modulus values of both groups (PNF only and PNF+PSA) and the individual changes. ^*^=significant change between pre- and post-values.

By analyzing the mean stiffness of the GM and GL, the ANOVA test revealed a significant time effect (*p*=0.05; *F*=4.45; *r*=0.46; *df*=17), but no group effect (*p*=0.26; *F*=1.37; *r*=0.27; *df*=17) or group×time interaction effect (*p*=0.051; *F*=4.4; *r*=0.45; *df*=17). A pairwise comparison of the shear modulus values of the gastrocnemius muscles showed a significant decrease in the PNF stretching+PSA group (*p*=0.0008; *d*=−0.71, medium effect), but not in the PNF stretching group (*p*=0.87; *d*=−0.04; Table 1; Figure 5).

### Maximum Voluntary Isometric Contraction Peak Torque

**Figure 6:**
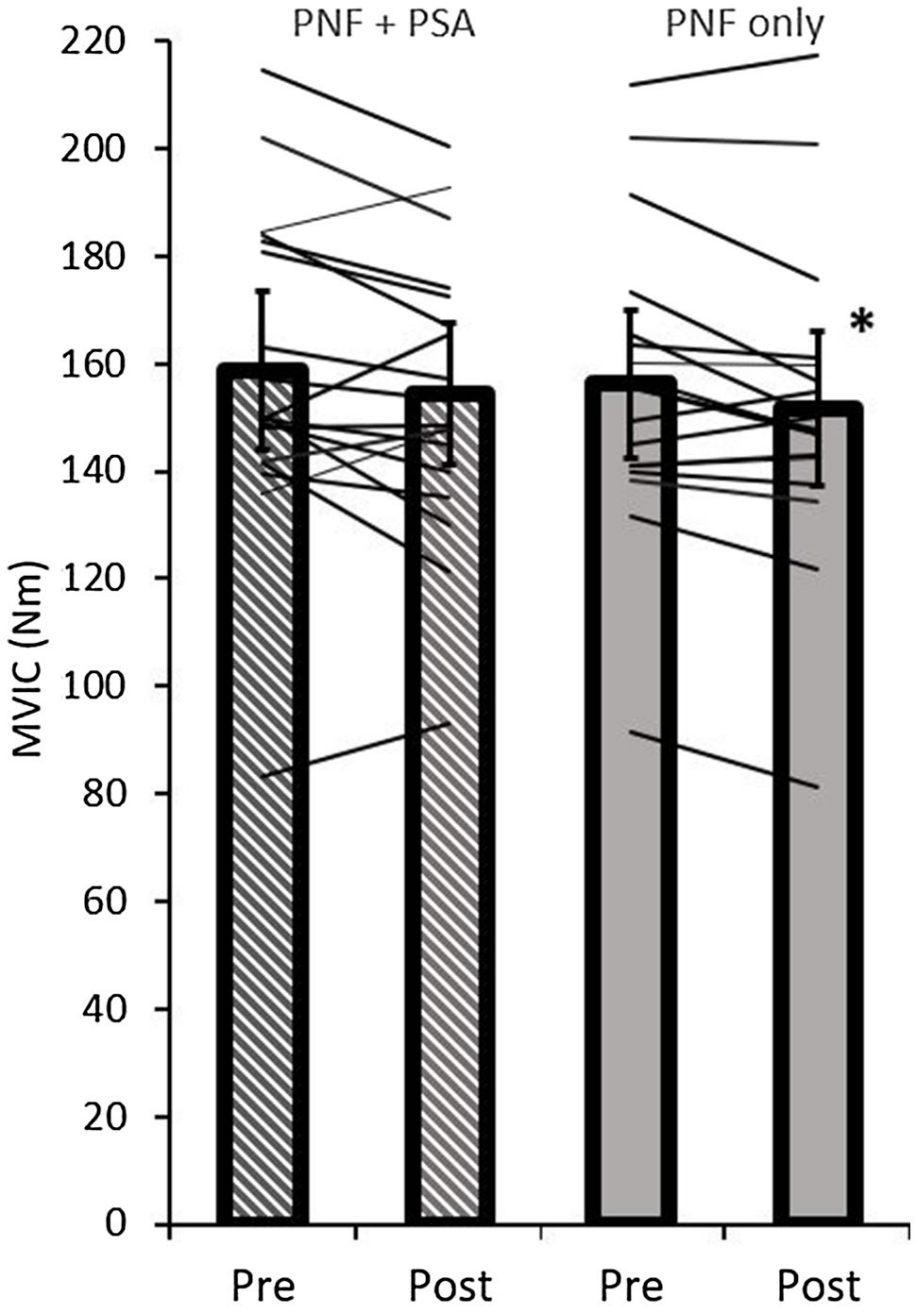
Pre- and post-MVIC peak torque values of both groups (PNF only and PNF+PSA) and the individual changes. ^*^=significant change between pre- and post-values.

The ANOVA test revealed a significant time effect (*p*=0.02; *F*=6.50; *r*=0.53; *df*=17), but no group effect (*p*=0.37; *F*=0.86; *r*=0.22; *df*=17) or time×group interaction effect on MVIC peak torque (*p*=0.91; *F*=0.01; *r*=0.03; *df*=17). A pairwise comparison showed a significant decrease in MVIC peak torque values in the PNF stretching group (*p*=0.01; *d*=−0.65, medium effect), but not in the PNF stretching+PSA group (*p*=0.11; *d*=−0.34; Table 1; Figure 6).

### Maximum Voluntary Dynamic Contraction Peak Torque

The ANOVA test revealed no significant time effect (*p*=0.16; *F*=2.14; *r*=0.33; *df*=17), group effect (*p*=0.54; *F*=0.39; *r*=0.15; *df*=17), or interaction effect (*p*=0.35; *F*=0.93; *r*=0.23; *df*=17; Table 1).

### Dorsiflexion ROM

**Figure 7:**
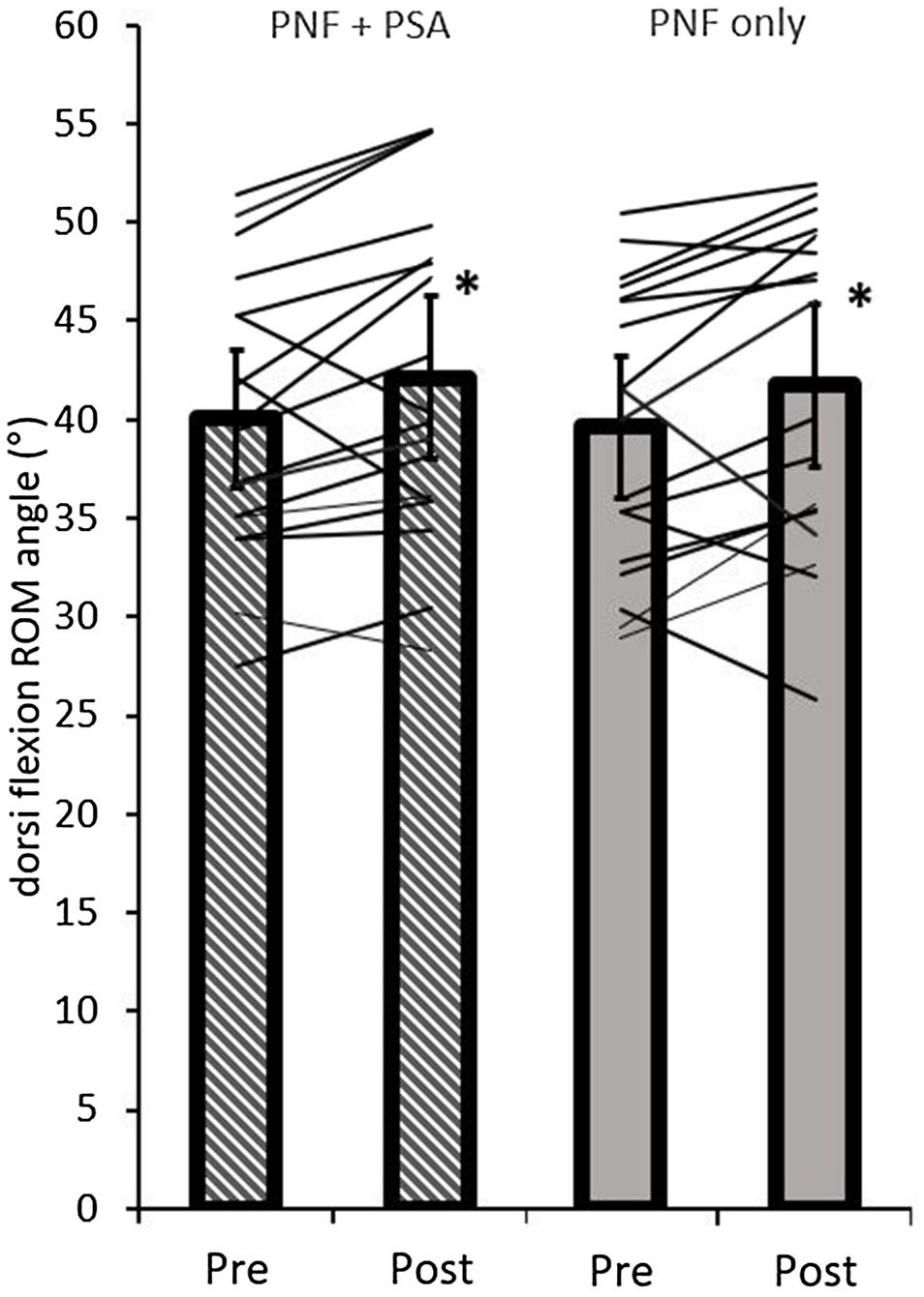
Pre- and post-dorsiflexion ROM values of both groups (PNF only and PNF+PSA) and the individual changes. ^*^=significant changes between pre- and post-values.

The ANOVA test revealed a significant time effect (*p*=0.002; *F*=12.80; *r*=0.66; *df*=17), but no group effect (*p*=0.83; *F*=0.05; *r*=0.05; *df*=17) or interaction effect (*p*=0.99; *F*=0.00; *r*=0; *df*=17). A pairwise comparison of the ROM values showed a significant increase in both groups, i.e., PNF stretching (*p*=0.04; *d*=0.53, medium effect) and PNF stretching+PSA (*p*=0.02; *d*=0.59, medium effect; Table 1; Figure 7).

### Passive Resistive Torque

The ANOVA test for PRT_max_ revealed no significant time effect (*p*=0.1; *F*=3.1; *r*=0.62; *df*=16), group effect (*p*=0.68; *F*=0.18; *r*=0.2; *df*=16), or group×time interaction effect (*p*=0.99; *F*=0.0; *r*=0.0; *df*=16; Table 1).

The ANOVA test for PRT at a given angle revealed no significant time effect (*p*=0.16; *F*=2.17; *r*=0.35; *df*=16), group effect (*p*=0.56; *F*=0.35; *r*=0.15; *df*=16), or interaction effect (*p*=0.25; *F*=1.41; *r*=0.28; *df*=16; Table 1).

### Correlation Analyses

Significant positive correlations between the delta values (post–pre) of ROM and PRT_max_ were found in both groups, i.e., PNF stretching (*p*<0.01; *r*=0.88; *n*=17) and PNF stretching+PSA (*p*<0.01; *r*=0.89; *n*=17).

A significant negative correlation between the delta values (post–pre) of ROM and shear modulus of GM (*p*=0.04; *r*=−0.5; *n*=17) was found in the PNF stretching group only, but not between the ROM and shear modulus of GL (*p*=0.84; *r*=0.06; *n*=17) or in the PNF stretching+PSA group.

## Discussion

The aim of the present study was to investigate the effects of a combination of PNF stretching and PSA on functional performance, ROM, and passive muscle stiffness, compared to isolated PNF stretching. The results of the study showed an increase in the dorsiflexion ROM of the ankle joint in both groups. However, only the combined intervention of PNF+PSA led to a decrease in muscle stiffness of the GM muscle and decreased mean muscle stiffness of the gastrocnemii. Furthermore, PNF+PSA did not change the MVIC peak torque values, while PNF stretching alone (without PSA) decreased MVIC peak torque significantly.

In some of the previous studies, it was found that the tested functional performance parameters (i.e., maximum muscle power output or strength values) decreased after a single PNF stretching exercise (Marek et al., 2005; Bradley et al., 2007; Konrad et al., 2017). On the other hand, Manoel et al. (2008) found no change in the dynamic muscle power output of the knee extensors after a single bout of PNF stretching in women, and the vertical jump performance values after PNF stretching did not change in the study of Christensen and Nordstrom (2008). Molacek et al. (2010) detected no change after a one repetition maximum test in the bench press in strength-trained football athletes. Although most of the studies found no change or a loss in functional performance parameters, an increase in muscle power parameters (counter movement jump, squat jump, and drop jump) after a single bout of PNF stretching was reported by Pacheco et al. (2011). The different findings might be the results of different stretching durations. A decrease in performance parameters was found after stretching durations of 2min or longer [Marek et al., 2005 (16min); Bradley et al., 2007 (10min); Konrad et al., 2017 (2min)]. Pacheco et al. (2011) stretched for 1min and found an increase in functional performance parameters, while Manoel et al. (2008) found no change after a stretching duration of 1.5min. The stretching duration of the present study was 2min, and therefore, a loss in performance parameter values in the PNF stretching group without subsequent PSA is in accordance with the previous findings. Most of the studies mentioned here measured muscle power performance in the lower extremities (i.e., leg muscle power performance during jumps or sprints). The present study measured the muscle strength in one muscle group only. Therefore, there are different system levels (e.g., the strength of one muscle group vs. the muscle power output of an extremity) included in these parameters, and a direct comparison is not possible. However, the findings of the different studies are pointing in the same direction and, in this case, there might be a connection between the measured levels. Therefore, in our opinion, it is possible to interpret the findings together, even if they occur on different system levels.

A possible explanation for the loss in muscle performance parameters might be the fact that the discussed findings involved high-intensity movements or maximal contractions (Hindle et al., 2012). Performance from submaximal movements, such as jogging, increased after a PNF stretching intervention (Hindle et al., 2012). The loss in muscle performance parameters at high intensities might be due to an inhibition in the muscle following a stretch greater than the capacity of the muscle (Hindle et al., 2012). Cramer et al. (2005) found a decreased EMG amplitude after static stretching, which might underline the inhibition theory of Hindle et al. (2012) for PNF stretching, even though the mechanomyography measurements (which measure and evaluate the low-frequency lateral oscillations of the activated muscle fibers) did not change (Cramer et al., 2005). Possible reasons for this might be neural factors, such as lower motor unit activation, firing repetition, or changed reflex sensitivity (Avela et al., 1999; Fowles et al., 2000; Behm et al., 2001). Another possible explanation might be the changed position of cross-bridges within the sarcomeres. In the lengthened position, and if continued for a long time, cross-bridges form in a stable way in a stretched sarcomere with reduced overlapping. If the muscle returns to the normal length, the rate of cross-bridge forming is reduced, and it takes some time to recover (Proske and Morgan, 1999). Another consideration according to the lower muscle power output is fatigue onset after the contractions during the PNF intervention, as proposed in Konrad et al. (2017).

In the present study, we found no change in MVIC peak torque values after the combination of PNF stretching+PSA. There is some evidence that PSA can counteract a decrease in performance parameters when applied following a single stretching exercise. For example, Reid et al. (2018) found a decrease in functional performance after 120s of static stretching, but the subsequent PSA of dynamic stretching/dynamic activity restored the functional performance parameters to baseline values. In contrast, static stretching durations for 30s and 60s led to no changes in MVIC and even an increased vertical jump performance. The added PSA therefore had a positive effect on all the parameters. These findings are in accordance with previous studies comparing the effect of PNF stretching durations on functional performance (Marek et al., 2005; Bradley et al., 2007; Manoel et al., 2008; Pacheco et al., 2011; Konrad et al., 2017). The loss in functional performance parameters after the PNF stretching but not after the PNF stretching+PSA observed in the present study is in accordance with the findings for other stretching techniques combining PSA (Reid et al., 2018). For example, Samson et al. (2012) found muscle power performance enhancing effects in sprint time after the combination of static or dynamic stretching and a sport-specific warm-up of the target muscle, while a general warm-up had no such effect. For this reason, the authors recommended PSA exercises activating the previously stretched muscle tissue, as applied in the present study. Another study showed that the order of the stretching and aerobic exercises in the warm-up plays an important role (Takeuchi et al., 2021). The authors found increases in ROM and stretch tolerance accompanied with decreases in (calculated) passive muscle-tendon stiffness after any combination (5min static stretching followed by 10min cycling on a cycle ergometer, 10min cycling followed by 5min static stretching, or 5min cycling+5min stretching+5min cycling). However, peak torque increases were only found when the aerobic exercises were performed after static stretching. The findings of this study indicate that PSA is a possible way to avoid functional performance output losses or even enhance functional performance following a single stretching exercise. In a more recent study, Takeuchi et al. (2021) found an increase in EMG values if PSA was performed after a static stretching intervention. However, it is still not clearly understood why PSA leads to such changes in functional performance parameters.

One possible explanation might be the reactivation of the conduction system of the nerves (Hindle et al., 2012) due to the specific movements activating the target muscles. Another possibility might be the reactivation of the energy storing capacity in the tendon. During the stretching process, the muscle-tendon unit becomes more compliant (Nakamura et al., 2011; Takeuchi et al., 2021), which may reduce its ability to store and release energy. The fast movements during PSA might reactivate the energy storage and transfer system due to increases in the elongation of the tendon during stretch-shortening movements as observed after a single exercise session (Hirayama et al., 2012) or after 12weeks of training (Kubo et al., 2017). Therefore, PSA might help to keep the power generating system intact, while the benefits of stretching, namely, a higher ROM, can also be maintained. Comparing the MVIC findings for the two groups in the present study, the possible fatigue due to the contractions following a PNF stretching exercise assumed by Konrad et al. (2017) is not a likely explanation. As the PNF stretching+PSA intervention included more contractions, it should have resulted in greater fatigue than PNF stretching alone. In contrast, PSA after PNF stretching prevented a possible loss in maximum force output. Therefore, the reduction in MVIC peak torque values after PNF stretching can likely be explained by the prolonged stretch of the sarcomeres and the arising changes in cross-bridge building during the stretching (Proske and Morgan, 1999), or the inhibition leading to a decrease in EMG amplitude (Cramer et al., 2005).

Another often-measured and performance-determining parameter is ROM. Similar to the findings of the present study, previous studies have reported an increase in ROM after a single PNF stretching exercise (Kay et al., 2015; Nakamura et al., 2015; Konrad et al., 2017). A possible explanation for the increased ROM might be changes in the formed cross-bridges during the stretching procedure (Proske and Morgan, 1999). During the prolonged lengthening in the maximum possible length of a muscle, new cross-bridges form that are quite stable. If the muscle is shortened back to the normal length, slack arises, and in combination with a reduced number of active cross-bridges, the passive muscle becomes more compliant (Proske and Morgan, 1999). Despite the ROM increases of 4% seen in both groups in the present study, the PRT values did not change at the maximum dorsiflexion ROM angles. Previous studies (Kay et al., 2015, 2016; Nakamura et al., 2015) reported interrelated increases in ROM and PRT_max_, which would indicate an increase in stretch tolerance (Magnusson et al., 1996). The present results were in contrast to these findings. Nevertheless, in both intervention groups of the present study (PNF stretching+PSA and PNF stretching alone), positive correlations between the changes in ROM and PRT were found, i.e., *p*<0.01, *r*=0.878 and *p*<0.01, *r*=0.892, respectively. This indicates changes in stretch tolerance as being a possible mechanism for the increase in ROM in both groups. While we did find a decrease in passive muscle stiffness in the PNF stretching+PSA group, no such changes were detected in the PNF stretching group. However, the changes in shear modulus were muscle-specific. We detected a significant difference in muscle shear modulus in the GM and the mean shear modulus (GM and GL), but not in the GL muscle after PNF stretching+PSA. Simpson et al. (2017) already reported differences in sehear modulus changes within a muscle group. They found different effects of stretching training in GM and GL fascicles and reported that changes in GM fascicles occurred faster than in GL fascicles. This might be a result of a more linear attachment of the GM fascicles to the tendon than in the GL. Therefore, it is assumed that during stretching more tension is applied to GM tissue than to GL tissue (Simpson et al., 2017). In combination with PSA, this might lead to the reduction in GM muscle stiffness but not in GL muscle stiffness in the present study. However, small but non-significant changes in GL stiffness were observed following PNF stretching+PSA. Therefore, it can be speculated that longer stretching or PSA durations and/or intensities might have also led to significant GL stiffness changes. This is supported by the analysis of the mean stiffness of the two gastrocnemii muscles, where we detected a decrease in stiffness after PNF stretching+PSA, but not after PNF stretching alone. A possible mechanism leading to a decreased passive muscle stiffness after PNF stretching+PSA could be an increase in the muscle-tendon unit temperature. Sapin-De Brosses et al. (2010) showed that a temperature rise in the muscle tissue is related to a stiffness decrease, which might explain the connection between PSA and the change in muscle shear modulus. Furthermore, there is a mechanical explanation. During movements, such as skipping or jumping, the muscle might be “stretched” eccentrically before the concentric contraction (stretch-shortening cycle) and hence the repetitive movements in the calf muscles (PSA) might intensify the stretching effect of the PNF stretching intervention. Another possible explanation might be changes in tendon tissue stiffness due to the stretch-shortening cycle. In the present study, only the muscle tissue was measured although there might also be acute changes in tendon tissue following the PNF stretching exercise (Kay et al., 2015). According to Kubo et al. (2017), fast movements might lead to higher acute tendon stiffness, which can result in decreased muscle stiffness. In contrast, Kubo and Ikebukuro (2019) analyzed muscle and tendon stiffness after repeated hopping exercises, and concluded that the acutely decreased joint stiffness is caused by changes in the active muscle stiffness, and not by changes in tendon properties. In the present study, in contrast to the findings of Kubo and Ikebukuro (2019), the PNF stretching prior to PSA might favor acute changes in the tendon stiffness.

## Conclusion

The results of this study showed that a single PNF stretching+PSA (2min stretching+two sets of three jumping and skipping exercises) intervention led to increased dorsiflexion ROM in the ankle joint and decreased muscle stiffness in the GM muscle and the mean muscle stiffness in the gastrocnemii, while the MVIC values did not change. In contrast, PNF stretching alone led to a decrease in MVIC values. Therefore, we conclude that, if PNF stretching is used as a warm-up exercise, target-muscle-specific PSA should follow to avoid possible performance losses while maintaining the benefit of greater ROM for increased flexibility in motion.

## Data Availability Statement

The raw data supporting the conclusions of this article will be made available by the authors, without undue reservation.

## Ethics Statement

The studies involving human participants were reviewed and approved by the ethical commission of the University of Graz. The patients/participants provided their written informed consent to participate in this study.

## Author Contributions

AK, MT, and MN collaborated in creating the concept of the study. The measurement methods were taught by GG and AA. MR was responsible for the data acquisition. The statistics were calculated in collaboration with MR and AK. MR, AK, MT, GG, AA, and MN collaborated in writing the manuscript. All authors read the final manuscript and approved the submitted version and also met the authorship criteria.

## Funding

This study was funded by a grant (Project P32078-B) from the Austrian Science Fund FWF.

## Conflict of Interest

The authors declare that the research was conducted in the absence of any commercial or financial relationships that could be construed as a potential conflict of interest.

## Publisher’s Note

All claims expressed in this article are solely those of the authors and do not necessarily represent those of their affiliated organizations, or those of the publisher, the editors and the reviewers. Any product that may be evaluated in this article, or claim that may be made by its manufacturer, is not guaranteed or endorsed by the publisher.
